# University presses, academic books, and authors in Ibero-America: a systematic review

**DOI:** 10.3389/frma.2026.1809274

**Published:** 2026-06-08

**Authors:** Sofia E. Calle Pesántez, Cristian Suárez-Giraldo, Alberto Ramos Alonso, Claudia Patricia Méndez-Rátiva, Adolfo A. Abadía

**Affiliations:** 1 Ibero-American Network for Scientific Studies on the Publishing, Evaluation and Circulation of Academic Books, Bogotá, Colombia; 2 Universidad de Salamanca, Salamanca, Spain; 3 Universidad Catolica de Oriente, Rionegro, Colombia; 4 Hipertexto Netizen, Bogotá, Colombia; 5 Universidad del Rosario, Bogotá, Colombia; 6 Universidad Icesi, Cali, Colombia

**Keywords:** academic books, Ibero-America, semantic networks and co-authorship, systematic review, university publishing

## Abstract

**Introduction:**

This study analyzes the literature on academic book publishing in Ibero-America indexed in Scopus and Web of Science, with the aim of describing its evolution and mapping co-occurrence and co-authorship networks to identify the current state of the field and existing gaps.

**Methods:**

A systematic review was conducted following the PRISMA 2020 protocol. Records were managed with Rayyan and analyzed using VOSviewer and R, incorporating thematic analysis of 44 selected studies.

**Results:**

The findings show a stronger concentration of publications in the last decade and an emerging field focused on editorial practices, open access, editorial professionalization, evaluation, and indicators. Co-authorship is concentrated in a few centers, and structural limitations persist that hinder robust comparisons.

**Discussion:**

The results highlight tensions in integrating books into scientific evaluation systems and in recognizing scientific development in areas of knowledge where books remain central. The study identifies gaps in the contextualization of evaluation frameworks that recognize the linguistic and publishing diversity of the Ibero-American region, calling for the consolidation of regulatory evaluation frameworks that strengthen scientific quality in academic publishing.

## 1 Introduction

Academic book publishing and the role of university presses are a central object of study for understanding the knowledge production and circulation processes in the Ibero-America region, encompassing the Caribbean and Latin American countries as well as Portugal and Spain. A quarter of the 21st century has already passed, and academic books continue to occupy a structural place in the region's scientific ecosystems. Despite repeated diagnoses of crisis or obsolescence, formulated from scientific communication models centered on the primacy of journals, the specialized literature recognizes that the academic book remains a relevant means of scientific communication. This especially applies in disciplines that require extensive and argumentative forms of exposition, such

as the humanities and social sciences. In this context, and in the face of digitization processes (Giménez-Toledo and Del Arco Blanco, 2022), academic books have adapted to contemporary scientific communication flows by incorporating digital formats, indexing processes, and online repositories, without losing their fundamental epistemological function (Spinak, 2018).

In the humanities and social sciences, books have historically been the preferred medium for extensive argumentation, theoretical development, and cultural contextualization of research results. These characteristics distinguish them from scientific articles and explain their persistence as academic communication in fields where knowledge construction requires analytical depth and interpretative density (Giménez Toledo, 2018). However, the hegemony of bibliometric indicators and the productivist orientation of scientific evaluation systems tend to undervalue this publication form, consolidating a structure of recognition that privileges indexed articles as the main measure of scientific impact, with negative effects on disciplinary and editorial diversity.

This tension is particularly visible in the Ibero-American context. The production of monographs and collective works by university or institutional publishers remains a common practice among researchers in the region. However, the visibility and value of these publications remain conditioned by the limited integration of academic books into international indexing systems, the concentration of their circulation in national or regional spheres, and the absence of standardized metrics that enable evaluating their impact in conditions comparable to other formats of scientific communication, especially journal articles (Hicks et al., 2015).

A turning point in the empirical understanding of this reality was the Cartography of Ibero-American Academic Publishing 2020–2021 (Giménez Toledo et al., 2020), the first systematic research aimed at mapping the production and circulation of academic books in the region. Based on an analysis of ISBN records for the 2013–2019 period, the study identified nearly 892,000 academic titles and located 465 active academic publishers. The findings revealed a strong geographical concentration of production, with six countries—Brazil, Spain, Mexico, Argentina, Colombia, and Chile—accounting for 91% of all registered academic titles (CERLALC et al., 2023). This project not only provided an unprecedented empirical basis for the size and structure of Ibero-American academic publishing but also established a methodological benchmark for analyzing the sector and designing policies on open science, bibliodiversity, and responsible knowledge assessment (FOLEC-CLACSO, 2023).

Despite these advances, significant analytical gaps persist in research on the Ibero-American academic publishing sector. Although there are relevant contributions from regional academic organizations and networks, such as the guidelines on responsible evaluation promoted by the Latin American Council of Social Sciences (CLACSO), sectoral reports of the Union of Spanish University Publishers (UNE), and structural studies of the Regional Center for Book Development in Latin America and the Caribbean (CERLALC), few comparative studies systematically integrate dimensions such as funding models, editorial quality and professionalization, the digital circulation of academic books, and the development of alternative impact metrics in line with disciplinary and regional specificities.

Within this framework, this systematic review aims to explore the profile of the agents who produce reflections on academic books in Ibero-America. It is pertinent to analyze whether these analyses mainly come from the strictly research sphere or, alternatively, whether a substantial part of the knowledge about the sector is generated from the professional practice of academic publishing. For this purpose, it is relevant to identify the extent to which university publishers, heads of institutional imprints, and other actors in the publishing ecosystem participate as authors in peer-reviewed academic publications or whether their contributions are mainly manifested through forms of knowledge production and circulation of a non-written nature, linked to practical and professional channels, such as specialized forums, book fairs, sector meetings, seminars, talks, oral presentations, or technical documents not subject to peer review.

From a conceptual standpoint, these approaches are part of the debates on bibliodiversity and cognitive sovereignty, which advocate the co-existence of multiple languages, publishing models, and epistemic approaches in scientific production (De Sousa Santos, 2014). In the face of global publishing concentration and the predominance of English as the hegemonic language of science, the Ibero-American academic book is emerging as a strategic space for cultural resistance and the defense of knowledge as a common good.

This position is conceptual and practical. One recent example is the *Guadalajara Agreement*, through which networks of Ibero-American university publishers, including the Association of University Publishers of Latin America and the Caribbean (EULAC), agreed to promote the recognition of Spanish and Portuguese as scientific languages, strengthen the circulation of academic publications in these languages, and coordinate joint actions for the publication and dissemination of open access knowledge in the region, thus reinforcing the bibliodiversity and cognitive sovereignty of local publishing systems (EULAC (Association of University Presses of Latin America and the Caribbean), 2023).

The need for a systematic review of the literature on academic books in Ibero-America responds to a twofold deficiency: on the one hand, the fragmentation of existing knowledge—scattered across disciplines, countries, and languages—and, on the other hand, the lack of coordination between theoretical studies and institutional policies that regulate scientific production, circulation, and evaluation. A well-structured systematic review enables addressing these gaps through analytical, critical, documentary, and propositional functions, aligned with the principles of open science and responsible evaluation (Hicks et al., 2015; FOLEC-CLACSO, 2022; UNESCO, 2021).

In this context, the need to promote inclusive, multilingual, and equitable scientific communication systems is particularly evident. Ibero-American academic books, supported by university and institutional publishers and open access policies, are a key tool for democratizing knowledge and strengthening regional intellectual sovereignty.

This systematic review is justified not only by its scientific value but also by its cultural and political dimension. The general objective is to analyze the research field on academic book publishing in Ibero-America based on literature indexed in international databases, aiming to construct contextualized indicators and conceptual frameworks, with four specific objectives:

- Review the scientific production addressing the central theme of academic book publishing in the Ibero-American region, published up to 2025, in the Scopus and Web of Science databases;
- Analyze thematic trends and temporal evolutions in the selected studies;
- Identify co-authorship networks and co-occurrence of terms that enable mapping the research field on academic books;
- Assess the strengths, limitations, and gaps in research on academic book publishing.

## 2 Method

**Figure 1:**
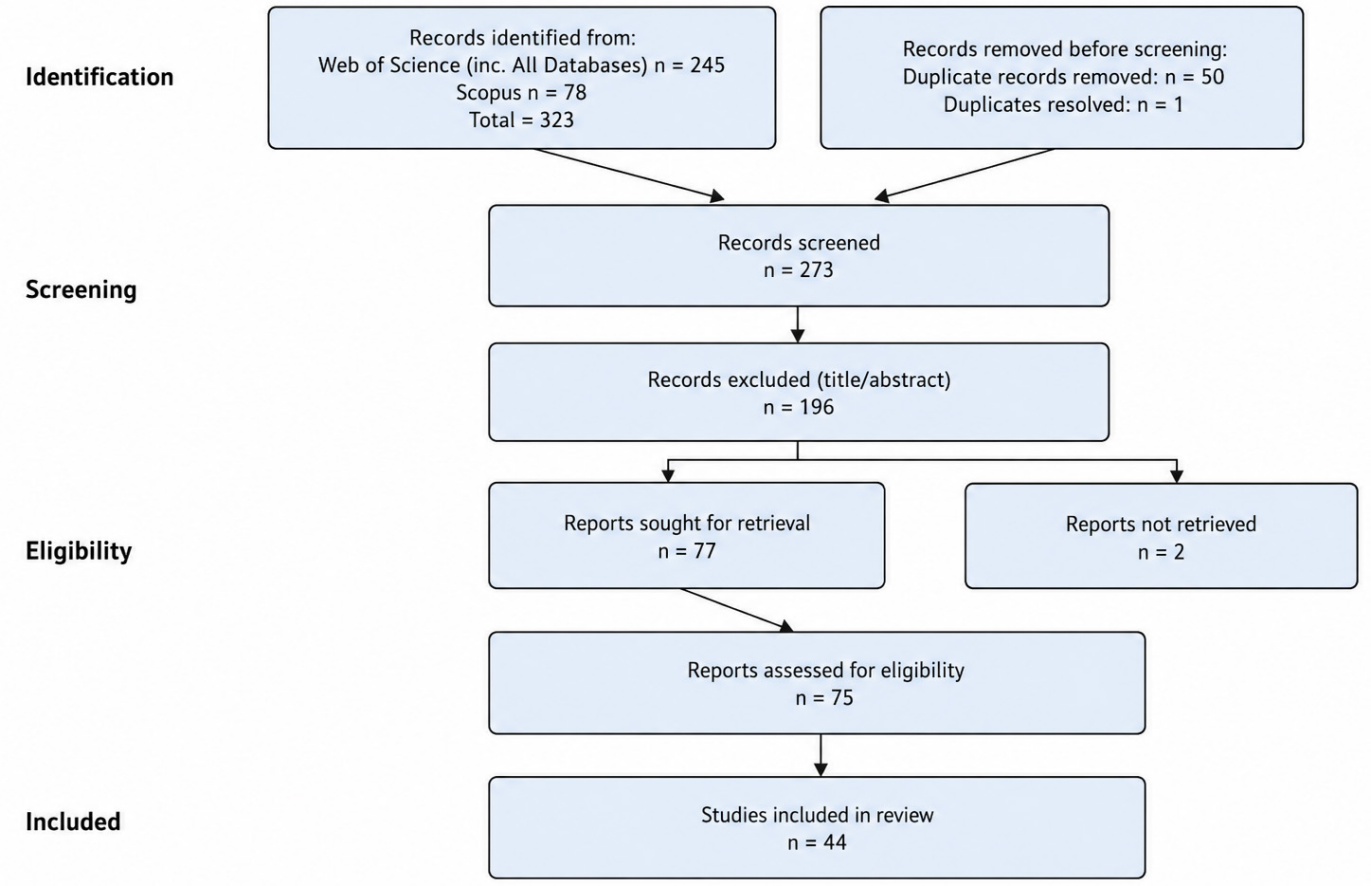
PRISMA 2020 flow diagram. Source: Own elaboration based on data analyzed with Rayyan.

This systematic review was developed following the Preferred Reporting Items for Systematic Reviews and Meta-Analyses (PRISMA) 2020 model. The study selection flow is reported using the flow diagram in Figure 1, based on the counts obtained from the review manager and the final extraction matrix.

### 2.1 Search strategy and information sources

The search was conducted without time restrictions, and the results were limited to documents in Spanish, English, and Portuguese. The Web of Science (all databases) and Scopus databases were consulted. Scientific articles, reviews, books, book chapters, and papers studying academic book publishing in Ibero-America (according to the geographical terms included in the search strategies) were considered eligible documents.

The search equations were as follows:

#### 2.1.1 Web of Science/All databases: 245 results

TS = (“academic book publishing” OR “scholarly book publishing” OR “academic book^*^” OR “scholarly book^*^” OR “academic monograph^*^” OR “scholarly monograph^*^” OR “book editing” OR “edición de libros académicos” OR “editorial universitaria” OR “editoriales universitarias” OR “editoriales académicas” OR “edição de livros acadêmicos” OR “editoras universitárias” OR “livros acadêmicos”)

AND

TS = (“Latin America” OR “South America” OR “Central America” OR “Caribbean” OR “Latin America” OR “Ibero-America” OR “Iberian America” OR “Hispanic America” OR “Argentina” OR “Bolivia” OR “Brazil” OR “Brazil” OR “Chile” OR “Colombia” OR “Costa Rica” OR “Cuba” OR “Ecuador” OR “El Salvador” OR “Guatemala” OR “Honduras” OR “Mexico” OR “México” OR “Nicaragua” OR “Panama” OR “Panamá” OR “Paraguay” OR “Peru” OR “Perú” OR “Dominican Republic” OR “República Dominicana” OR “Uruguay” OR “Venezuela” OR “Spain” OR “España” OR “Portugal”)

#### 2.1.2 Scopus: 78 results

TITLE-ABS-KEY (“academic book publishing” OR “scholarly book publishing” OR “academic book^*^” OR “scholarly book^*^” OR “academic monograph^*^” OR “scholarly monograph^*^” OR “book editing” OR “edición de libros académicos” OR “editorial universitaria” OR “editoriales universitarias” OR “editoriales académicas” OR “edição de livros acadêmicos” OR “editoras universitárias” OR “livros acadêmicos”)

AND

TITLE-ABS-KEY (“Latin America” OR “South America” OR “Central America” OR “Caribbean” OR “Latin America” OR “Ibero-America” OR “Iberian America” OR “Hispanic America” OR “Argentina” OR “Bolivia” OR “Brazil” OR “Brazil” OR “Chile” OR “Colombia” OR “Costa Rica” OR “Cuba” OR “Ecuador” OR “El Salvador” OR “Guatemala” OR “Honduras” OR “Mexico” OR “México” OR “Nicaragua” OR “Panama” OR “Panamá” OR “Paraguay” OR “Peru” OR “Perú” OR “Dominican Republic” OR “República Dominicana” OR “Uruguay” OR “Venezuela” OR “Spain” OR “España” OR “Portugal”)

A total of 323 references were imported into the screening manager (Rayyan).

### 2.2 Eligibility criteria

#### 2.2.1 Inclusion criteria

- Empirical or theoretical studies analyzing the publication of academic books by universities or academic publishers in Ibero-America.
- Documents with data on circulation, visibility, impact, publishing policies, metadata management or collaboration networks, editorial quality, identifiers, publication models, distribution, and marketing that consider academic books in their study samples.
- Studies on science evaluation focused on academic books.

#### 2.2.2 Exclusion criteria

- Studies focused on formats other than academic books.
- Publications not directly related to academic book publishing in Ibero-America.
- Brief notes or opinions without systematic analysis.
- Types of documents other than scientific articles, reviews, books, book chapters, and papers.

### 2.3 Study selection and screening process

References were managed in the Rayyan screening environment for duplicate detection and staged refinement. Of the 323 records initially imported, 51 duplicate records were identified; 50 were removed and one was resolved by retaining the main record, leaving 273 unique records for screening. Screening was conducted in two phases:

- First, the 273 unique records were screened by title and abstract according to the predefined inclusion and exclusion criteria. In this phase, 196 records were excluded and 77 reports were sought for retrieval.
- Second, full-text evaluation was conducted for these 77 reports. Of these, 75 full texts were successfully retrieved and assessed for eligibility, whereas 2 reports could not be accessed. After full-text assessment, 31 reports were excluded for not meeting the inclusion criteria, and 44 studies were included in the final review. The main reasons for exclusion at the full-text stage were lack of thematic relevance, lack of geographical relevance to Ibero-America, and mismatch with the object of study.

### 2.4 Data extraction

Extraction was performed using a structured matrix in Excel. For each study included, the institution or publisher analyzed or affiliated with, type of document, country or region addressed by the article, objective(s) of the study, methodological design, techniques or tools used, key results or findings, main topics addressed (coded), indicators or metrics mentioned, structural or contextual factors identified, gaps or limitations of the study, conceptual or practical contribution, and recommendations or implications proposed by the authors were systematically recorded.

### 2.5 Synthesis and analysis strategies

The synthesis of results was conducted by combining descriptive analysis with a narrative synthesis structured by thematic categories. First, a basic quantitative characterization of the corpus (temporal trends, geographical distribution, and types of documents) was developed based on the variables extracted in the matrix. Second, the interpretation of the field was strengthened by network analysis, including co-authorship networks and semantic networks based on the co-occurrence of terms extracted from the keywords and processed in VOSviewer, as well as the creation of N-grams from the abstracts using RStudio.

For the network analysis of the 44 documents included in this review, first all information was standardized based on its inclusion in the Zotero bibliographic manager. Second, considering that the review included documents written in Spanish, English, and Portuguese, the English versions of the titles, abstracts, and keywords were used to standardize the bibliographic information. For the book by Dujovne et al. (2025), a translation was chosen due to the unavailability of data in English. Finally, a file was exported from Zotero in.RIS format for the construction of networks in VOSviewer and.CSV format for the creation of two- and three-word N-grams in R.

Third, a narrative synthesis was conducted in five analytical categories that structure the presentation and interpretation of the findings: (i) university and regional publishing practices, (ii) open access and visibility, (iii) evaluation and indicators, (iv) publishing management and professionalization, and (v) internationalization and circulation of knowledge. This organization enabled integrating heterogeneous results and comparing approaches, emphases, and gaps between lines of research within the field.

## 3 Results

Overall, the results show an expanding field of study, especially in the last decade, with a growing interest in the characterization, professionalization, and digital transformation of university publishing, albeit marked by methodological, conceptual, and structural heterogeneities.

**Figure 2:**
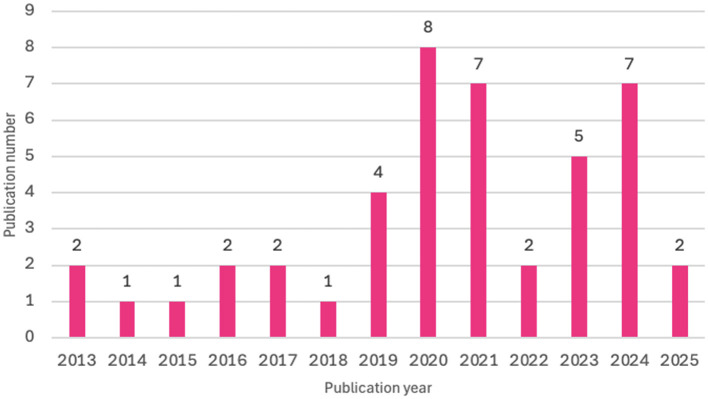
Annual number of publications on academic book publishing in Ibero-America. Source: Own elaboration based on the analyzed data.

The basic quantitative characterization showed that the production of publications related to academic book publishing in Ibero-America is growing, but in a very incipient way, with the scientific article format predominating. Figure 2 shows the production volume per year of the analyzed corpus. In there, two moments are identified, one from 2013 to 2018, with around 1 or 2 publications per year, and another from 2019 to 2025, with a much broader production and a highest point in 2020 with 8 published documents.

**Figure 3:**
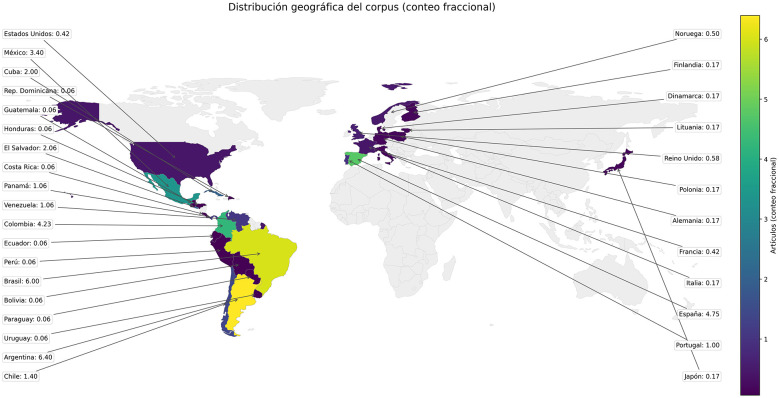
Geographical distribution of the bibliographic corpus (fractional counting). Source: Own elaboration based on the analyzed data.

The geographical coverage of the corpus was estimated using a fractional count to avoid overestimating countries mentioned in comparative articles. In this scheme, each article contributes a total weight of 1 distributed among the countries covered. The resulting map in Figure 3 summarizes the geographical distribution of equivalent articles by country, with Argentina, Brazil, Spain, Colombia, and Mexico leading the ranking. Four articles in the sample analyzed do not report an identifiable geographical area.

### 3.1 Co-authorship and semantic co-occurrence networks

In the co-authorship network, each node represents an individual present in a publication, and its size is determined by the frequency of documents in which they participate as an author. The edges are the links connecting a pair of nodes and accounting for a co-authorship relationship. In the co-occurrence network, each node represents a term used as a keyword in the documents reviewed, and the link is generated by its presence alongside other keywords in the documents. The size of the node is defined by the frequency of the term's occurrence. In both cases, it is an undirected network, namely, the relationship between nodes is not only determined by a place of origin and a place of arrival (directed), but rather both actors are mutually connected in a bidirectional manner.

**Figure 4:**
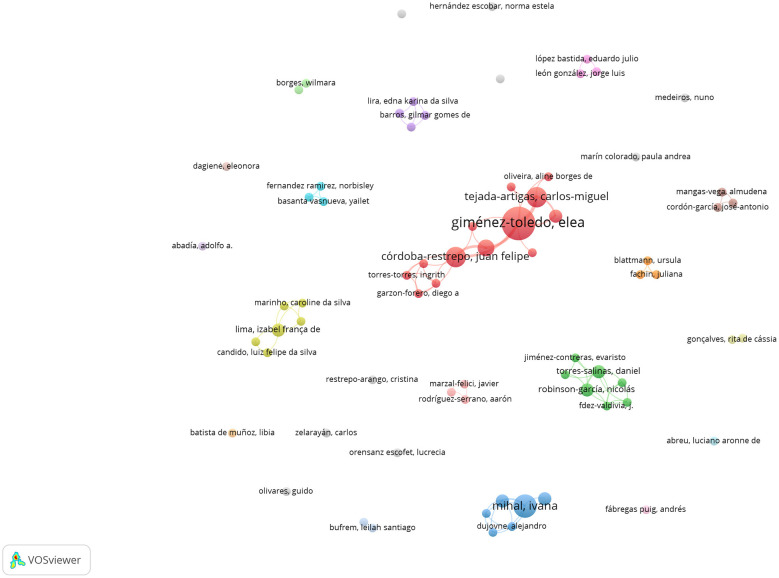
Co-authorship network (69 nodes/80 edges/27 clusters). Source: elaboration based on data analyzed with VOSviewer. Interactive network: https://tinyurl.com/23ygezfw.

Figure 4 shows that isolated contributions predominate in the scientific output of the corpus, corresponding to authors with a single published article, some of them co-authored with two, three, and even four collaborators. This limits the density of the network and the cohesion among researchers in the field. Disregarding these nodes or subgroups, three clusters can be identified, each with a more relevant node depending on the area it covers. These three clusters of actors are recognized by red (13 nodes), green (7 nodes), or blue color (6 nodes) because they constitute subgroups of more than four actors.Authors occupying central positions in each cluster can be identified. Ivana Mihal is the central author of the blue cluster with five documents addressing university and academic publishing as a cultural, political, and strategic practice, based on the study of public reading policies and their relationship with the publishing market and marketing models, the digital mediatization of publishers' communication, and the valuation of academic books in scientific evaluation systems.

With seven nodes, the green cluster has two dominant authors: Daniel Torres-Salinas and Nicolás Robinson-García, who are present in the publication of two articles focused on the scientometric study of book citation patterns, the influence of publishers on the visibility of knowledge, and the main differences according to disciplinary areas.

The largest and most diverse cluster in terms of contributors to academic production is the red one, which revolves around Elea Giménez-Toledo, who is also the most prolific author in this study (10 articles). This forms a central line of study of the academic book as the central object of the scientific communication system in the social sciences and humanities, from conceptual and methodological perspectives that characterize the role and prestige of publishers in the evaluation, visibility, and institutional recognition of academic books and editors.

This subgroup of nodes is an example of relational capital and continuity in the work of authors such as Giménez-Toledo and Mihal, who mobilize social, economic, and academic resources to form collaborative working groups that are beginning to stand out, for example, from an analysis of social networks such as this one. Within the main cluster (red), other authors are emerging, such as Juan Felipe Córdoba Restrepo, Carlos-Miguel Tejada-Artigas (with 5 articles each), and Esteban Giraldo-González (with 4 articles). Such authors play other key roles within this subgroup of authors by attracting new nodes and, quite possibly, outlining complementary lines of work from comparative analyses of academic publishing in Ibero-America with an emphasis on publishing actors, scientific collaboration, and book production, aiming to identify the bases of evaluation and prestige of publishers as a key factor in the scientific communication system.

**Figure 5:**
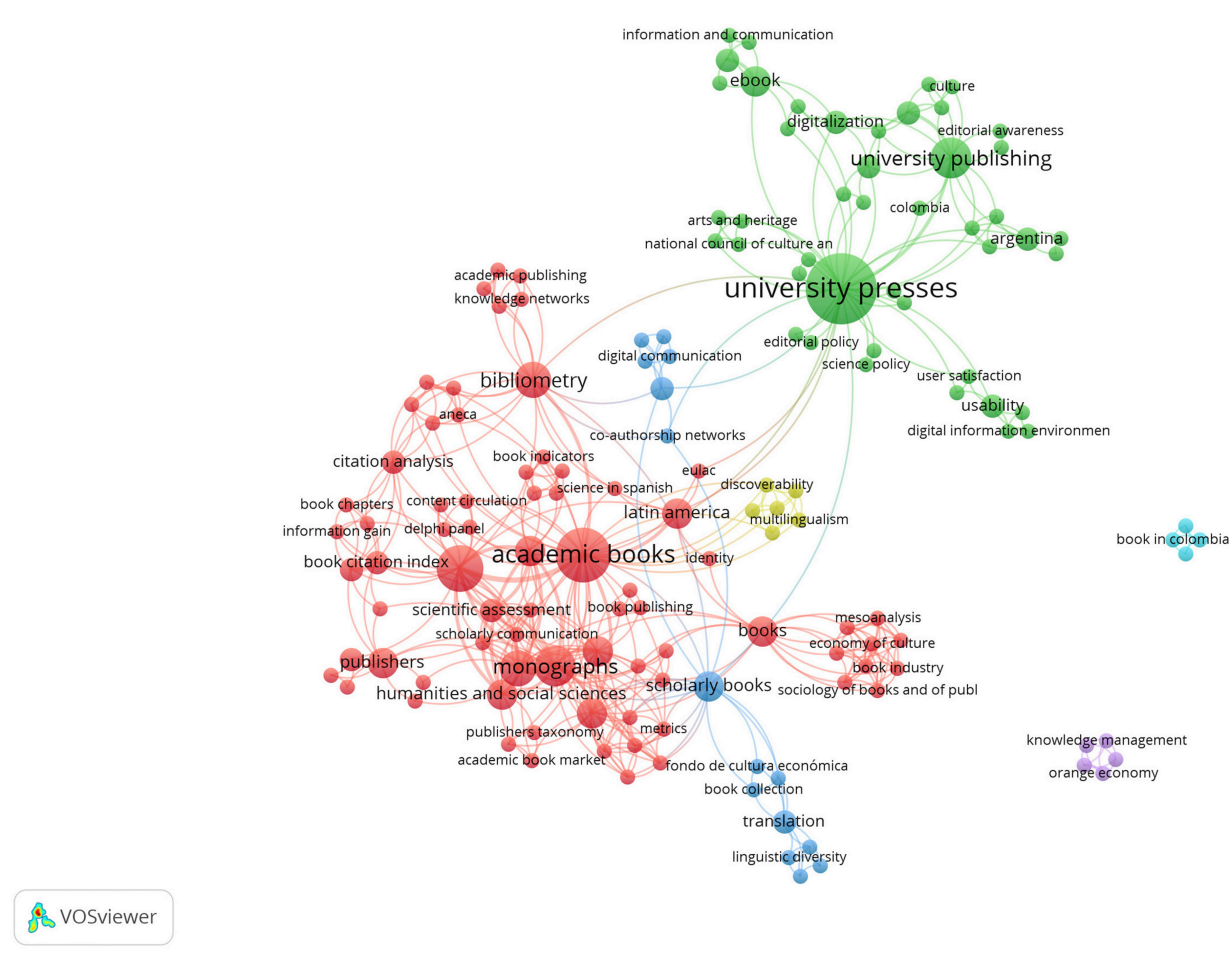
Semantic co-occurrence network (150 nodes/497 edges/7 clusters). Source: elaboration based on data analyzed with VOSviewer. Interactive network: https://tinyurl.com/2879fhet.

The co-occurrence network in Figure 5 shows a configuration of word groups that centers around two main axes: *academic books* (red cluster) and *university presses* (green cluster). Between these two large clusters lies a third cluster (blue) whose central node is the keyword *scholarly publishing*. The figure shows four additional clusters, which are not located on the periphery or isolated from the larger set of connected nodes (*giant component*) that comprises the three aforementioned clusters.

Note that each cluster comprises a subgroup of nodes that could well constitute lines of work in themselves. In response to the red cluster, topics associated with academic books are prioritized based on book types (*monographs, textbooks, and book chapters; scientific assessment of books: scientific assessment, scholarly communication, content circulation*, and *quality*), as well as production measurement (*bibliometrics, metrics, citation analysis*, and *book indicators*). Other terms are noted that could shape new trends in the study of academic books, such as *academic book market, sciences in Spanish, Latin Americ*a, and *book industry*.

The green cluster groups words around one of the key players in academic book production: *university presses*. Here, we find words that bring to mind both editorial management: *digitalization, editorial awareness, e-books, editorial policy*; the georeferential configuration of university presses: Argentina, Portugal, and Colombia; and references to the research ecosystem in which universities are embedded: *science policy, national council, culture*, and *scientific publishing*. Other keywords are found at the extremes of this subset of nodes, but might gain relevance as university presses continue to exploit their capacity for innovation through new digital resources, social networks, and, more recently, artificial intelligence, as suggested by terms associated with *information* and *communication, digital information environment, user satisfaction, and usability*.

The third cluster is found in the concept of scholarly publishing, although it could also be established alongside the term scholarly books as reference points, from which what could be understood as a series of studies framed within specific analytical approaches emerge. These can be understood as innovative ventures that emerge from the mainstream but are also distinct from it, including terms such as *co-authorship networks, linguistic diversity, translation, books collection*, and even *social network analysis*.

It is worth mentioning that the first and third clusters share the term *books*, but the complete configuration of the concepts *academic books* and *scholarly books* tends to be used almost interchangeably as synonyms. This led to the consideration of constructing bigrams and trigrams of terms to identify other possible configurations, which would be worth establishing as equivalents or further exploring their differentiation in future studies, or even considering the formalization of a specialized thesaurus on this subject.

**Figure 6:**
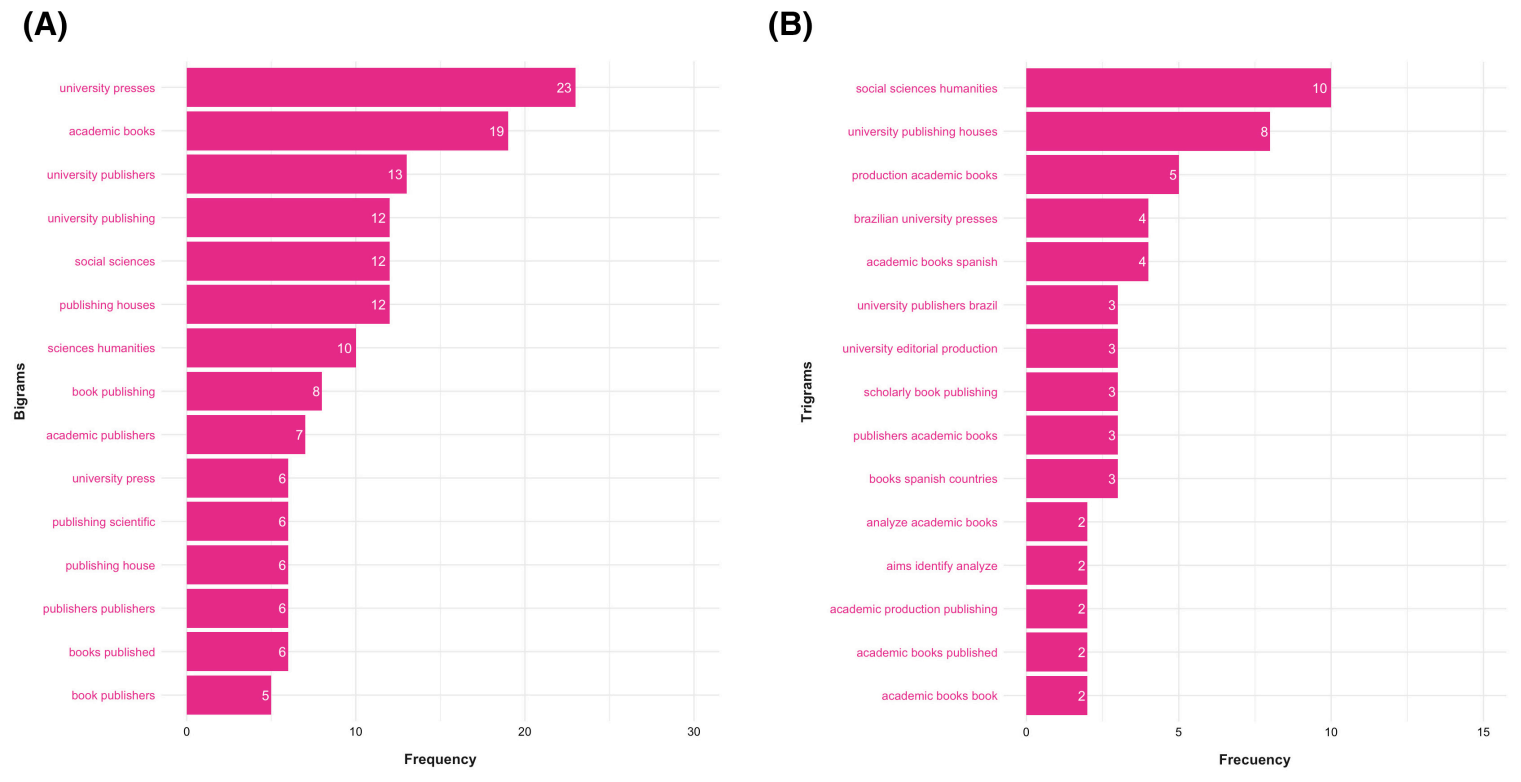
N-gram frequencey **(A, B)**. Source: Own elaboration based on data analyzed with R.

**Figure 7:**
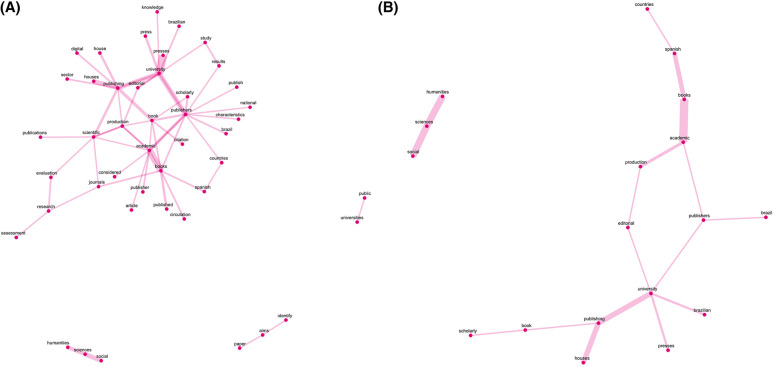
N-grams network **(A, B)**. Source: Own elaboration based on data analyzed with R. Both graphs have the minimum frequency set at 2.

Consequently, to complement this semantic analysis, N-grams were constructed, and the frequency of concepts composed of two and three words was identified, complemented with graphs, such as Figures 6, 7. To collect a broader sample of terms, the abstracts in English of the reviewed documents were taken. Accordingly, Figure 6 illustrates the 15 most prominent terms, as determined by the number of words comprising the bi- or tri-word concept. Figure 7 presents the relations between lexical units and the intensity of two-word relations, thereby facilitating the discernment of lexical usage and semantic understanding implemented in the literature reviewed. Here, the thickness of the linkages corresponds to the frequency of the pairings.

When detailing the *bigrams*, the most recurrent concept is *university presses*, followed by *academic books*, corresponding to the semantic network in Figure 5. However, closely related configurations begin to emerge, such as *university publishers, university publishing, academic publishers*, university *press(es), publishing house(s), book publishers*. There are also two concepts that are not synonymous but are very present in studies of academic book production (Giménez-Toledo, 2020; Giménez Toledo and Córdoba Restrepo, 2018; Tejada-Artigas et al., 2020), namely, social *sciences* and *humanities*.

Looking at the trigrams, the most frequent term is connected just as we closed the previous paragraph, referring to a combination of three words to form the concept of *social sciences humanities*. Combinations such as *scholarly book publishing, academic production publishing, and university editorial production* begin to emerge, as well as concepts such as *publisher academic books, production academic books, and academic book published*, which refer to the units that produce books and to the book product itself. Unlike what is reported in the top 15 bigrams, this list of concepts composed of three terms begins to offer clues to other thematic lines of work that determine a place, region, or language, such as *Brazilian university presses, academic book Spanish, books Spanish countries*, as well as a kind of methodological approach and disciplinary contribution, such as *methodology study qualitative* and *programs information science*.

The semantic mapping shows that academic books and university presses are the two poles that organize the literature reviewed, albeit not always in unambiguous or clearly defined relationships. While academic books emerge as multiple objects—evaluated, measured, translated, and contextualized disciplinarily and geographically—university presses are configured as spaces for management, innovation, and cultural mediation embedded in national and international scientific ecosystems.

The co-existence of terms used interchangeably in clusters, bigrams, and trigrams, together with the emergence of more specific conceptual configurations, particularly in trigrams, suggests an expanding field, traversed by tensions between standardization and linguistic diversity, between traditional and emerging methodological approaches. Additionally, it highlights the structural interdependence between academic books and university publishers and the absence of fully consolidated terminological and conceptual consensus, which opens avenues for further exploration of these definitions and approaches by the group of authors who have published on this topic and by new generations of scholars in areas related to academic books and university publishers.

### 3.2 University and regional publishing practices

There is a preeminence of studies dedicated to reviewing the specific modes of production, selection, circulation and legitimization of academic books in specific university and territorial contexts, with case studies and comparative analyses of Ibero-American academic publishing being particularly noteworthy.

Thus, countries such as Portugal (Medeiros, 2015), Cuba (León González et al., 2016; Fernández Ramírez et al., 2019), Chile (Olivares, 2020), Panama (Batista de Muñoz, 2021), El Salvador (Hernández Escobar, 2022), and Venezuela (Borges and Rodríguez Acasio, 2023) are studied individually, mainly from a descriptive perspective of the current situation and the prospects and challenges of publishing management. The approaches to these countries are characterized by proposing publishing management models adapted to political, institutional, and professional circumstances.

On the one hand, countries such as Argentina (Dujovne, 2020; Zelarayán, 2020; Szpilbarg, 2020; Mihal, 2021; Vicente Romero, 2021), Brazil (Bufrem and Freitas, 2018; De Lima et al., 2018; Giménez-Toledo et al., 2019; De Abreu, 2019; Barros et al., 2024), Colombia (Marín Colorado, 2020; Giraldo-González et al., 2022; Restrepo-Arango, 2023; Abadia, 2023; Méndez-Rátiva et al., 2025), Mexico (Guerra González, 2016; Mihal, 2024; Giraldo-González et al., 2024a), and Spain (Giménez-Toledo et al., 2013; Cordón-García et al., 2019; Marzal-Felici et al., 2021; Calleja Ibáñez and Giménez-Toledo, 2024) are not only addressed by a larger number of studies, but also indicate that university publishing practices face common challenges related to professionalization, economic sustainability, and adaptation to digital environments. Despite this, studies agree in recognizing their fundamental contribution to the diversity of the academic publishing ecosystem and to the circulation of knowledge that would otherwise struggle to find a place in commercial or international circuits.

However, comparative studies point, in general terms, to the discrepancy between the academic value of books and their recognition in evaluation systems, the urgent need for digital transformation and professionalization of the publishing sector, the importance of networking to overcome limitations, the tension between regional identity and international standards, and the requirement for specific policies that recognize the particularities of regional academic publishing (Giménez-Toledo et al., 2015; De Oliveira and de Cássia Gonçalves, 2016; Bufrem and Freitas, 2017; Magadán-Díaz and Rivas-García, 2020; Tejada-Artigas et al., 2020; Mihal and Szpilbarg, 2020; Giménez Toledo et al., 2020; Vázquez Álvarez, 2021; Fábregas Puig, 2021; Giraldo-González et al., 2024b; Mihal, 2024; Mihal and Saferstein, 2024).

### 3.3 Open access and visibility

Studies highlight the growing role of digital books and open access as mechanisms for expanding circulation and visibility. Advances have been identified in the adoption of digital platforms, such as OMP (Barros et al., 2024), institutional repositories, and online catalogs. However, limitations persist in relation to the interoperability of information systems, the standardization of metadata, and the absence of clear international distribution strategies (Blattmann et al., 2020; Restrepo-Arango, 2023). Furthermore, there is a recognized need to strengthen digital communication policies in the sector, support smaller publishing structures through partnerships, and consider medialization as a strategic dimension of the publishing field in future scenarios (Mihal and Saferstein, 2024).

While open access has established itself as a central axis for the visibility and democratization of knowledge, driven by both institutional initiatives and public policies aimed at open science, studies show that the implementation of these policies presents uneven developments among countries and institutions (Barros et al., 2024). In several contexts, open science policies lack specific guidelines for academic books, favoring scientific articles and leaving regulatory gaps in funding models, evaluation, and incentives for open access book publishing (Giménez Toledo and Córdoba Restrepo, 2018; Dujovne et al., 2025). However, studies in Argentina (Zelarayán, 2020; Mihal, 2021) and Chile (Olivares, 2020) highlight the importance of state support for the promotion of reading, through which university books have achieved greater circulation. This situation creates tensions in relation to the real conditions of Ibero-American publishing.

In relation to the economics and sustainability of academic books, the results indicate that the expansion of open access has deepened the financial challenges that university publishers face (Marín Colorado, 2020). The reduction in revenue from book sales has not been systematically offset by alternative financing mechanisms, increasing the dependence on institutional subsidies and public funds. The studies analyzed agree on the absence of consolidated economic models that coherently integrate open access, editorial quality, and financial sustainability.

They also highlight the difficulties of internationalization due to technological limitations, recommending strengthening online purchasing and marketing channels, the increased adoption of open access, and improved digital visibility and metadata for publishing catalogs (Restrepo-Arango, 2023).

### 3.4 Evaluation and indicators

There is a significant effort to analyze the university publishing landscape using indicators that measure, above all, the production of academic books, as well as their distribution and availability. The authors mainly determine the number of academic or university publishers through reports from scientific evaluation agencies, research systems, or databases and indexing. Works including Giménez-Toledo et al. (2015), Cordón-García et al. (2019), Giménez-Toledo (2020), Tejada-Artigas et al. (2020), and Marzal-Felici et al. (2021) show a correlation between the number of researchers, the registration of university publishers, and the publication of books and chapters visible in information systems to highlight the volume of Ibero-American book production and the main subjects of this scientific communication medium. However, Dagiene (2023) offers the most integrated comparison of national models for evaluating books and recommends adding new approaches to book evaluation that consider all stages of the publishing process.

One indicator of analysis was the ISBN registry, used to quantitatively or bibliometrically contrast a country's total book production with total national publishing production, as in the case of Mexico (Giraldo-González et al., 2024a) and Colombia (Giraldo-González et al., 2022), or comparatively among Latin American countries (Giraldo-González et al., 2024b). However, the evidence from this approach has demonstrated the need to train publishers in the correct registration of metadata with the Book Chambers, establish consensus on the definition of the book and academic publishing (Giraldo-González et al., 2024c), and strengthen dialogue between the different actors in the publishing ecosystem to promote the circulation of academic books.

In addition, one predominant finding is that the assessment of academic publishers has been based predominantly on reputational models, supported by interviews or qualitative inquiries with researchers and publishers. Studies including Giménez-Toledo et al. (2013), Giménez-Toledo et al. (2019), Tejada-Artigas et al. (2020), Giménez Toledo et al. (2021), and Dujovne et al. (2025) show that the quality and prestige of Ibero-American university publishing are based on aspects such as track record, thematic specialization, the technical quality of the works, and the authors' experience with these institutions.

It is also worth highlighting studies conducted in relation to the Book Citation Index, which analyze the citation behavior of academic chapters and books, allowing us to understand the role of different types of publishers in citation indices (Torres-Salinas et al., 2013, 2014). Although these studies show a geographical bias toward English-language publishers, they offer useful conceptual and methodological tools for studying academic publishing and its patterns of impact, providing a framework applicable to the analysis of university publishers and academic books in Ibero-America.

### 3.5 Publishing management and professionalization

One of the main issues addressed in the studies corresponds to university publishing management and professionalization in Ibero-America, where structural challenges are shared that indicate a field in the process of strengthening (Vázquez Álvarez, 2021). These include the need for greater alignment with universities' institutional projects, the strengthening of transparent and visible publishing processes, and the consolidation of professional capacities to ensure quality, sustainability, and consistency with the mission of academia (Medeiros, 2015; Zelarayán, 2020; Restrepo-Arango, 2023).

The main challenges identified include the lack of clear criteria and transparent information on editorial evaluation processes, which generates mistrust among researchers; the weakness in the specialized training of editors, associated with the absence of accredited programs; and institutional imbalances related to funding, distribution, and digital visibility. These limitations affect the adoption of electronic formats, proper metadata management, and indexing on specialized platforms, thereby impacting the visibility and impact of publishing catalogs.

In response, proposed strategies are aimed at strengthening explicit publishing policies, adopting management models based on processes and performance indicators (Batista de Muñoz, 2021; Méndez-Rátiva et al., 2025), and continuous professionalization through publishing networks and associations. Likewise, the importance of advancing external peer review (Dujovne et al., 2025), decisive migration to digital environments, and diversification of sources of sustainability (Restrepo-Arango, 2023) would improve the quality, academic reputation, and visibility of Ibero-American university publishers, contributing to open access and bibliodiversity (Zelarayán, 2020).

### 3.6 Internationalization and circulation of knowledge

The studies grouped in this category address the issue regarding the circulation of academic books, publishing prestige, translation mechanisms, participation in international markets, and the use of digital platforms.

A first set of studies relates to the visibility of academic production, where it is evident that although books continue to be a relevant means of scientific communication—especially in the social sciences and humanities—their impact and recognition remain lower than those of indexed scientific articles (Marzal-Felici et al., 2021). Some comparative studies show that books have slower and more localized circulation, although they play a key role in the transfer of contextualized knowledge and in languages other than English.

Regarding the prestige and recognition of academic publishers, the results indicate that institutional reputation and membership of recognized catalogs significantly influence authors' perceptions of quality and publication decisions. However, regional asymmetries have been identified, with Latin American university publishers enjoying lower levels of international recognition than European and North American publishers, despite the academic quality of their content (Torres-Salinas et al., 2013).

Another relevant area is editorial translation. Some studies document pioneering experiences in translation and co-publishing that have enabled expanding the circulation of Latin American academic works, although they highlight that such initiatives tend to depend on specific projects rather than systematic publishing policies (Orensanz Escofet, 2017; Giménez Toledo, 2020; Mihal, 2024). Therefore, translation appears to be a strategic factor.

Some studies analyze the participation of university presses in book fairs and international markets, showing that although these spaces favor visibility and exchange, effective circulation and tend to be concentrated in a small number of titles and publishers with greater commercial and logistical capacities (Mihal, 2021). In this context, the work of associations and networks, such as the Association of University Publishers of Colombia (ASEUC), the Brazilian Association of University Publishers (ABEU), the Network of University and Academic Publishers of Mexico - Altexto, and EULAC, is key to integration, dissemination, distribution and technical-scientific strengthening, with successful dissemination and distribution strategies (De Oliveira and de Cássia Gonçalves, 2016).

## 4 Discussion

### 4.1 Quantitative characterization and geographical distribution

The temporal evolution of the publications analyzed suggests that the study of university publishing and academic books in Ibero-America remains a relatively nascent field, albeit with signs of consolidation over the last decade. In the sample analyzed, production has grown particularly in the last decade, mainly concentrated on the scientific article format (Figure 2). This pattern is consistent with a transition from specific contributions or case studies to a more systematic agenda, driven by the complementary needs to (1) characterize the university publishing ecosystem (management, professionalization and digital transformation) and (2) build comparable instruments for evaluating books, publishers, and their circulation.

In this vein, the international impetus comes from debates on responsible evaluation and the limits of traditional indicators, which have reopened the discussion on how to adequately recognize products other than articles, such as academic books (Hicks et al., 2015). More specifically, efforts are being made to measure and recognize publishers and books in the humanities and social sciences, which has led to methodological proposals aimed at evaluating scientific publishers and reviewing national and international initiatives (Giménez-Toledo et al., 2013, 2015; Giménez Toledo, 2018). At the same time, the incorporation of sources such as the Book Citation Index and its bibliometric analysis has helped position the topic, although it has also revealed coverage biases and interpretation challenges for books and publishers (Torres-Salinas et al., 2013, 2014).

Regarding geographical distribution, estimation using fractional counting is methodologically relevant because it avoids over-representing countries that appear only as part of comparisons. Under this scheme, the map in Figure 3 summarizes “equivalent articles” by country. With this approach, Argentina, Brazil, Spain, Colombia, and Mexico appear as the main centers. Rather than interpreting this as a direct reflection of “where” university publishing occurs, this result shows where research and publication on the topic have taken place in the sources consulted. This concentration might be associated with the presence of active research communities, more coordinated university publishing networks, and the greater availability of data and measurement initiatives in those countries; for example, studies focused on Brazil or Colombia on publishing prestige and researchers' perceptions (Giménez-Toledo et al., 2019; Tejada-Artigas et al., 2020). At the same time, the existence of articles without an identifiable geographical area suggests the co-existence of more conceptual or international contributions, which, although valuable for constructing comparative frameworks, make it difficult to attribute the knowledge produced to a specific territory.

### 4.2 Co-authorship and semantic co-occurrence networks

Two articles in the literature review analyze co-authorship networks (Abadia, 2023; Méndez-Rátiva et al., 2025). Although each article focuses on a particular university press, their results share elements of collaboration analysis that correspond with this study in at least three points.

The first relates to the configuration of a highly fragmented network. Accordingly, in the configuration of the global co-authorship network, authors who report low production, either individually or with few collaborators, co-exist alongside others who denote high production and collaboration with other researchers. According to Abadia (2023), this reflects poorly consolidated collaboration networks and a dispersed collaborative fabric (Méndez-Rátiva et al., 2025) in the field of university presses and academic books. Second, without ignoring the above, this low density in the link between nodes does not translate into a lack of collaboration between subgroups of authors, who have managed to establish a collaborative working community with more stable dynamics of association in scientific production. Third, the centrality of certain individuals within these communities, or clusters, suggests a leadership position within their subgroup.

Although these communities have been established based on joint authorship of scientific products, these documents contain information that would allow us to establish thematic associations of this production, in which we could identify coincidences in theoretical and methodological approaches, the definitions proposed, and the key terminology used.

In relation to the co-occurrence analysis of terms conducted in this study, and in line with the above, the idea of fragmentation is further explored, now understood as conceptual dispersion around academic book and university press as the key concepts of the field. Both the semantic network and the N-grams constructed reveal the existence of multiple terms used to refer to similar issues, confirming the lack of homogeneous terminology. This finding reinforces Giraldo-González et al.'s (2024c) statement regarding the difficulty of establishing stable and consensual definitions in the field of academic publishing.

In this sense, the conceptual dispersion identified constitutes not only an analytical limitation but also an opportunity that would allow progress toward the construction of coherent descriptions and shared definition criteria, aimed at strengthening the theoretical foundations of the field and enabling the realization of comparable studies, in which the central concepts are used with greater precision and without ambiguity. Accordingly, greater homogenization would contribute to consolidating the conceptual maturity of studies on academic books and university presses.

### 4.3 Narrative synthesis and analytical categorization

While the literature reviewed on academic publishing, university presses, and academic books shows progress in characterizing the field, it reveals structural and methodological limitations that restrict the explanatory scope of the studies. According to the results, problems are identified associated with the absence of standardized databases or sources of information for academic books, the dependence on outdated secondary sources, deficiencies in selection and registration, standardization and harvesting of metadata, especially ISBNs, by authors and publishers, and incomplete geographical coverage of countries such as Peru, Bolivia, Paraguay, Uruguay, Ecuador, Nicaragua, and Costa Rica, which affects the robustness of comparative analyses and the generalization of results. These weaknesses condition the holistic understanding of the university publishing ecosystem, particularly in Ibero-American contexts.

In relation to *university and regional publishing practices*, studies tend to work with incomplete catalogs, inaccurate historical data, and analyses focused on isolated case studies. This limits the possibility of understanding structural dynamics, such as marketing models, financial sustainability, or relationships between actors in the academic book ecosystem. The discussion suggests that the field requires comparative research—both quantitative and qualitative—that moves beyond purely descriptive approaches by incorporating interviews, analysis of publishing processes, and regional impact assessments. From this perspective, an imperative consequence is to foster inter-institutional and international alliances that allow the collection of bibliographic data under open and accessible governance and infrastructure models.

Regarding *open access and visibility*, the literature agrees that incomplete digitization of catalogs, low-quality metadata, and heterogeneous institutional policies hinder effective knowledge circulation. Although open access is recognized as a strategic principle, its implementation in the field of academic books presents regulatory and technical gaps, especially in relation to e-books, multimedia formats, and sustainable financing models. In this regard, highlights the need for studies that evaluate specific policies for academic e-books and regional visibility platforms, as well as their real impact on the use and circulation of content. In addition, opportunities such as the recent European Accessibility Act are emerging for academic publishers to structure book publishing with a perspective that transcends electronic formats and focuses on the social reach of knowledge.

Furthermore, university publishers must take advantage of and use open access digital book platforms, such as the Directory of Open Access Books (Ferwerda et al., 2023) and Dialnet, which have seen considerable growth in the downloading of academic books, or online publishing catalogs, such as Ulibros de EULAC, Unilibros by ASEUC, Unebook by UNE, and Altexto, to name a few, which, under a hybrid model, enrich the availability of bibliographic information, expand book sales channels, and favor institutions that lack their own e-commerce channel. In this panorama of options for accessing academic publishing, platforms such as Hipertexto-Netizen with its SIMEH metadata management application and online libraries such as eLibro or Digitalia have also positioned themselves, strengthening the commercial dimension of e-books and the range of titles with a wider geographical reach.

In terms of *evaluation and indicators*, persistent biases have been identified arising from the use of citation metrics dominated by Anglo-Saxon bases, such as the Book Citation Index or Google Scholar. The absence of verifiable criteria and disciplinary weightings limits the proper assessment of academic books, especially in the social sciences and humanities, restricting the measurement of impact on citation. It is suggested to move toward hybrid evaluation models that integrate alternative indicators aligned with principles such as DORA or ICEE, combining quantitative and qualitative approaches that recognize the epistemological diversity of academic books. In this regard, national systems and institutional policies on the recognition of researchers' output should consider new approaches to assessment.

The category of *editorial management and professionalization* highlights the lack of organizational transparency, the absence of accredited editorial training, and the limited systematization of processes through performance indicators as one of the most critical gaps in the field. The few studies available also reflect institutional weaknesses in the generation and disclosure of information. In this context, the discussion highlights the urgent need to strengthen networks, publishing observatories, and process management models, as well as to promote public policies aimed at professionalizing the university publishing profession. From this perspective, the recent creation of the Ibero-American Council for University and Academic Publishing (CIEUA) at the 2025 Guadalajara Book Fair becomes an important mechanism for coordinating these efforts (CLACSO, 2025).

Finally, the *internationalization and circulation* of knowledge appear to be limited by the lack of multilingual studies, the scant attention paid to translation processes, and the inconsistency of the global data available. The predominance of national or regional approaches restricts understanding of the international trajectories of academic books. Consequently, the literature suggests broadening the research agenda to include comparative global analyses, debates on multilingualism, and international frameworks for the evaluation and circulation of knowledge, such as those proposed by CoARA or the Helsinki Declaration.

Overall, the field of university publishing is in a phase of conceptual consolidation but still faces significant challenges in terms of data quality, methodological approaches, and international coordination. Addressing these gaps is key to strengthening future research and positioning the academic book as a central object in science, higher education, and knowledge circulation policies.

## 5 Conclusions

This systematic review enabled characterizing the scientific production indexed in Scopus and Web of Science up to 2025 on academic book publishing in Ibero-America, consolidating an updated overview of a field that, although still limited in volume, shows growth and progressive structuring. The findings confirm that academic books remain a central object for understanding regional scientific communication, especially in the social sciences and humanities, although the challenges of international visibility, dominant scientific evaluation frameworks, and uneven digital transformation processes continue to strain their study. There has been a greater concentration of publications in the last decade, coinciding with an emerging agenda driven by debates on responsible evaluation and the need to build comparable instruments to recognize and analyze books, publishers, and their circulation. Likewise, the thematic analysis identified five main areas of research: university and regional publishing, open access and visibility, evaluation and indicators, publishing management and professionalization, and internationalization and circulation. For these reasons, it can be said that this article fulfilled the objectives outlined in the introduction.

Regarding the configuration of the field through networks, the co-authorship analysis shows that research is concentrated in a few authorial nuclei, with relatively stable clusters and a high proportion of authors with occasional participation. This pattern is consistent with a field in consolidation that still depends on small communities and specific leaderships. For its part, the term co-occurrence network confirms that concepts linked to *academic books* and *university presses* articulate the dominant semantic core, while emerging categories such as linguistic diversity, translation, alternative metrics, and digital platforms are beginning to delineate recent lines with potential for expansion. However, the co-existence of similar expressions or expressions used as synonyms reinforces the idea of conceptual dispersion and low terminological standardization, which hinders the comparative accumulation of knowledge and opens up an opportunity to strengthen conceptual consensus and shared definitions in future research.

One element that warrants critical attention based on the results is the relative scarcity of publications detected in Scopus and Web of Science on academic book publishing in Ibero-America. Rather than being interpreted as a lack of experience or professional development in the publishing field, this finding raises the relevant research question concerning whether professional communities linked to academic publishing produce little systematic writing in general, or if they produce knowledge that fails—or does not seek—to enter these international databases. In this sense, the absence of records in these databases could reflect a gap between publishing practice and indexed scientific publication, where those who actively participate in the production and validation of knowledge remain relatively invisible as authors within formal academic communication systems.

Similarly, it is plausible that a significant part of editorial knowledge circulates in formats and spaces not captured by Scopus and Web of Science—conferences, book fairs, professional networks, institutional workshops, internal documents, or local repositories—where learning and operational solutions are shared without necessarily being transformed into products that meet international indexing criteria. In this sense, the problem would not only be a “lack of writing,” but also a disconnect between the circulation of publishing knowledge and channels of academic visibility, influenced by workload, a lack of incentives, and the applied nature of the profession. Consistent with this, the corpus located is mainly oriented toward bibliometric and evaluative approaches (visibility, indicators, and internationalization), while the lack of works on everyday publishing tasks—metadata, interoperability, distribution, digital circulation, standards, or sustainability—suggests that these practical dimensions are dealt with in non-indexed areas. Therefore, the production visible in Scopus and WOS offers a partial overview of the field, focusing more on its evaluative performance than on strengthening the publishing profession.

In this context, the decision to focus on Scopus and Web of Science responds to the aim of analyzing the internationally visible and indexed scientific production, enabling the construction of a standardized, comparable, and reproducible corpus in line with PRISMA guidelines. Rather than aiming to exhaustively cover all forms of editorial knowledge production, this study deliberately concentrates on the segment of the field that is formally recognized within global systems of academic evaluation. This approach allows for a consistent analytical framework while explicitly acknowledging that it captures only one dimension of a broader and more heterogeneous ecosystem.

This possible “invisibility” of publishing output in international databases should not be interpreted solely as an individual deficit or a shortcoming of the field, but rather as an indicator of how recognition, time, and legitimate formats for knowledge production are distributed. In this context, a future research agenda could focus on mapping alternative circuits of writing and circulation of editorial knowledge, contrasting indexed databases with regional repositories, conference proceedings, technical manuals, institutional documents, or professional literature. In this way, the challenge would not only be to increase the volume of scientific publications but also to understand where, how, and for whom editorial knowledge is written in Ibero-America, and what conditions would enable its greater documentation, accumulation, and visibility.

Finally, some future lines of research can be envisaged, including studying the impact of commercial academic publishers that offer specialized services to researchers (with APC charges, for example) on Ibero-American university publishing, as a recent phenomenon that not only questions the economic model of university publishers but also their purpose and scope as units for the transfer of knowledge from universities. Along the same lines, understanding the dynamics of the commercial circulation of academic books holds relevance, as well as multichannel and multiformat commercial distribution in academic publishing houses. However, there is a clear need to consolidate and structure data on academic publishing, compiling bibliographic information to support scientific evaluation processes and policies on science, technology, and innovation. In addition, it would be worthwhile to generate university publishing management models adapted to innovation in information systems, digital processes, and media, developments in artificial intelligence, and new conceptions of the role of the publisher in the context of scientific evaluation.

From these findings, several practical and policy-oriented implications emerge. For university presses, the results highlight the need to strengthen the documentation and systematization of editorial practices—particularly in areas such as metadata management, interoperability, and digital circulation—to enhance both operational efficiency and scholarly visibility. For research evaluation agencies, the study underscores the importance of broadening recognition frameworks beyond internationally indexed outputs, incorporating diverse formats and regional channels where editorial knowledge is produced and circulated. At a policy level, fostering infrastructures that integrate indexed and nonindexed production—such as regional repositories and collaborative data systems—could contribute to reducing asymmetries in visibility and supporting a more inclusive model of scientific communication.

## Data Availability

The datasets generated and analyzed for this study are available in the Zenodo repository at https://doi.org/10.5281/zenodo.18603247.

## References

[B1] Abadia A. A. (2023). Co-authorship networks and scholarly books. a methodological approach from a university press case study. CS J. 40, 103–142. doi: 10.18046/recs.i40.5858

[B2] Barros G. G. de Lira, E. K. da S. Miranda A. C. D. Jacintho EMDSB (2024). University publishers in Brazil: use of OMP software for editing, mapping of privacy policies, accessibility and use and reproduction licenses. Rev. Digit. Bibliotecon. Ci. Inf. 23:e025004. doi: 10.20396/rdbci.v23i00.8677145

[B3] Batista de Muñoz L. (2021). Management of university presses and the strategic transfer of knowledge in Panama. SIGNOS Res. Manag. Syst. 13:2. doi: 10.15332/24631140.6665

[B4] Blattmann U. Fachin J. Werlang E. (2020). Perspectives of the academic open access e-book. Rev. Ibero-Am. Cienc. Inf. 13, 522–547. doi: 10.26512/rici.v13.n2.2020.21154

[B5] Borges W. Rodríguez Acasio F. (2023). University publishing production in northern venezuela: perspectives, realities and challenges. InterMeio Rev. Prog. Pós-Grad. Educ. UFMS 29:57. doi: 10.55028/intermeio.v29i57.18697

[B6] Bufrem L. S. Freitas J. L. (2017). University editors and scientific information: rethinking the university editor. Brazilian Research Trends in Information Science, 10:1. Available online at: https://revistas.ancib.org/tpbci/article/view/433 doi: 10.36311/1981-1640.2016.v10n2.11.p89 (Accessed June 21, 2024).

[B7] Bufrem L. S. Freitas J. L. (2018). University editors and scientific information: rethinking the university editor. Tend. Pesqui. Bras. Ciênc. Inf. 10:1. Available online at: https://revistas.ancib.org/tpbci/article/view/433 (Accessed June 21, 2024).

[B8] Calleja Ibáñez P. Giménez-Toledo E. (2024). Exploring named-entity recognition techniques for academic books. Learned Publ. 37:e1610. doi: 10.1002/leap.1610

[B9] CERLALC CSIC, EULAC, and SEGIB. (2023). Cartography of Ibero-American Academic Publishing. Regional Center for the Promotion of Books in Latin America and the Caribbean. Available online at: https://cerlalc.org/publicaciones/cartografia-de-la-edicion-academica-iberoamericana/ (Accessed May 12, 2026).

[B10] CLACSO (2025). Ibero-American Council for University and Academic Publishing. https://www.clacso.org/consejo-iberoamericano-de-edicion-universitaria-y-academica/ (Accessed February 21, 2025).

[B11] Cordón-García J.-A. Merchán-Sánchez-Jara J. Mangas-Vega A. (2019). Evolution of the visibility of scholarly monographs in the academic field. El Prof. Inf. 28:4. doi: 10.3145/epi.2019.jul.09

[B12] Dagiene E. (2023). Prestige of scholarly book publishers–an investigation into criteria, processes, and practices across countries. Res. Eval. 32, 356–370. doi: 10.1093/reseval/rvac044

[B13] De Abreu L. A. (2019). Academic training and production: the role of university editors. Ibero-Am. Stud. 45, 163–173. doi: 10.15448/1980-864X.2019.2.32339

[B14] De Lima I. F. de Lima R. F. da Silva Marinho C. de Medeiros Villar e Silva H. C. (2018). Endorsing the usability of two websites of Brazilian university publishers. Ci. Inf. Rev. 5, 42–53. doi: 10.28998/cirev.2018v5n2d

[B15] De Oliveira R. C. de Cássia Gonçalves R. (2016). University editors in Latin America: the importance of work in associations. Rev. Ciênc. Hum. 17, 39–59. doi: 10.31512/rch.v17i28.2146

[B16] De Sousa Santos B. (2014). Epistemologies of the South: Justice Against Epistemicide. London: Routledge.

[B17] Dujovne A. (2020). Gutenberg attends in Buenos Aires: university publishing in the face of the geographical concentration of the Argentine publishing market. *Cuad. Cent. Estud. Diseñ. Comun*. 85, 35–47. doi: 10.18682/cdc.vi85.3750

[B18] Dujovne A. Mihal I. Saferstein E. Bonacci J. M. Ostroviesky H. (2025). Books and Their Value in Scientific Evaluation: Analysis and Proposals Based on the Argentine Case. Córdoba, Argentina: Editorial Universidad Católica de Córdoba.

[B19] EULAC (Association of University Presses of Latin America and the Caribbean), (2023). EULAC and Ibero-American publishing cooperation. Available online at: https://eulac.org/2024/12/las-editoriales-universitarias-iberoamericanas-firman-el-acuerdo-de-guadalajara (Accessed Febuary 28, 2019).

[B20] Fábregas Puig A. (2021). The book: a factor in Latin American/Caribbean identity. AKADEMOS, 13, 7–12. doi: 10.5377/akademos.v0i0.11634

[B21] Fernández Ramírez N. Basanta Vásnueva Y. Cabrera Morales I. (2019). Two years from the visibility strategy for Ediciones Universidad de Camagüey. *Rev. Publicando* 6, 23–33. Available online at: https://revistapublicando.org/revista/index.php/crv/article/view/1700 (Accessed March, 2026).

[B22] Ferwerda E. Snijder R. Stern N. (2023). Open access to books—the perspective of a non-profit infrastructure provider. J. Electron. Publ. 26:3303. doi: 10.3998/jep.3303

[B23] FOLEC-CLACSO (2022). A New Academic and Scientific Evaluation for a Socially Relevant Science in Latin America and the Caribbean: Declaration of Principles. Buenos Aires: CLACSO. Available online at: https://biblioteca-repositorio.clacso.edu.ar/bitstream/CLACSO/169563/1/Declaracion-CLACSO-FOLEC-version-extendida.pdf (Accessed May 12, 2026).

[B24] FOLEC-CLACSO (2023). Declaration of the Latin American Forum on Scientific Evaluation: For an open and bibliodiverse science. Buenos Aires: CLACSO. Available online at: https://biblioteca-repositorio.clacso.edu.ar/bitstream/CLACSO/169563/1/Declaracion-CLACSO-FOLEC-version-extendida.pdf (Accessed February 9, 2026).

[B25] Giménez Toledo E. (2018). Research assessment in Humanities and Social Sciences in review. Rev. Esp. Doc. Cient. 41:e208. doi: 10.3989/redc.2018.3.1552

[B26] Giménez Toledo E. (2020). Why books are important in the scholarly communication system in social sciences and humanities. Sch. Assess. Rep. 2:6. doi: 10.29024/sar.14

[B27] Giménez Toledo E. Córdoba Restrepo J. F. (eds.). (2018). Academic Publishing and Dissemination: Open Books in Ibero-America. Bogotá: Editorial Universidad del Rosario; Editorial Comares. doi: 10.12804/th9789587841671

[B28] Giménez Toledo E. Córdoba Restrepo J. F. García Valencia E. (2020). Cartography of Ibero-American Academic Publishing. Available online at: https://pti-esciencia.csic.es/project/cartografia-de-la-edicion-academica-iberoamericana (Accessed May 12, 2026).

[B29] Giménez Toledo E. Córdoba Restrepo J. F. Giraldo González E. Mañana Rodríguez J. (2021). Quality and prestige in academic publishing: the Colombian case. Signo Pensam. 40:78. doi: 10.11144/Javeriana.syp.40-78.cpea

[B30] Giménez-Toledo E. (2020). Why books are important in the scholarly communication system in social sciences and humanities. Schol. Assess. Rep. 2:6. doi: 10.29024/sar.14

[B31] Giménez-Toledo E. Del Arco Blanco A. (2022). Digitization of Academic Publishers. Granada: Editorial Comares.

[B32] Giménez-Toledo E. Mañana-Rodríguez J. Tejada-Artigas C.-M. (2015). Review of national and international initiatives on books and book publishers' assessment. El Prof. Inf. 24:705. doi: 10.3145/epi.2015.nov.02

[B33] Giménez-Toledo E. Tejada-Artigas C.-M. Borges-De-Oliveira A. (2019). Books and academic publishers according to Brazilian researchers in social sciences and humanities. Inf. Prof. 28:6. doi: 10.3145/epi.2019.nov.03

[B34] Giménez-Toledo E. Tejada-Artigas C.-M. Manana-Rodriguez J. (2013). Evaluation of scientific books' publishers in social sciences and humanities: results of a survey. Res. Eval. 22, 64–77. doi: 10.1093/reseval/rvs036

[B35] Giraldo-González E. García-Valencia E. Córdoba-Restrepo J. F. Giménez-Toledo E. (2024a). Publishers and production of academic books in Mexico: 2013–2019. Eur. Sci. Edit. 50:e123288. doi: 10.3897/ese.2024.e123288

[B36] Giraldo-González E. Giménez-Toledo E. Córdoba-Restrepo J. F. (2022). Production of academic books in Colombia between 2013 and 2019: a step forward for comparative studies. Investig. Bibl. 36:153. doi: 10.22201/iibi.24488321xe.2022.93.58633

[B37] Giraldo-González E. Giménez-Toledo E. Córdoba-Restrepo J. F. (2024b). The production of academic books in 16 Latin American countries between 2013 and 2019. Rev. Esp. Doc. Cient. 47:e389. doi: 10.3989/redc.2024.1520

[B38] Giraldo-González E. Giménez-Toledo E. Córdoba-Restrepo J. F. (2024c). What is an academic book publisher? An Ibero-American contribution to the definition. Insights 37:1. doi: 10.1629/uksg.669

[B39] Guerra González J. T. (2016). The publishing activity of the National Autonomous University of Mexico. *Cuad. Investig. Cienc. Inf*. 1, 11–38. doi: 10.34295/cuinci.vi1.5

[B40] Hernández Escobar N. E. (2022). Evolution and challenges of university presses in El Salvador in the democratization of knowledge. Sci. Cult. Soc. 7, 83–92. doi: 10.5377/ccs.v7i2.14496

[B41] Hicks D. Wouters P. Waltman L. de Rijcke S. Rafols I. (2015). Bibliometrics: the leiden manifesto for research metrics. Nature 520, 429–431. doi: 10.1038/520429a 25903611

[B42] León González J. L. López Bastida E. J. Mora Quintana E. C. (2016). Toward a new model of university publishing at the University of Cienfuegos. *Univ. Soc*. 8, 186–192. Available online at: https://rus.ucf.edu.cu/index.php/rus/article/view/486 (Accessed March, 2026).

[B43] Magadán-Díaz M. Rivas-García J. I. (2020). Disruptive impact of e-book on Spanish publishers' value chain: a case study. Rev. Esp. Doc. Cient. 43:e258. doi: 10.3989/redc.2020.1.1650

[B44] Marín Colorado P. A. (2020). The book in Colombia: between sustained concentration and the slow march toward independence (2000–2019). Amoxtli 5, 39–58. doi: 10.5281/zenodo.4377459

[B45] Marzal-Felici J. Rodríguez-Serrano A. Soler-Campillo M. (2021). Comparison of the impact of books and articles by Spanish communication researchers through Google Scholar in 2019. Rev. Esp. Doc. Cient. 44:e288. doi: 10.3989/redc.2021.1.1744

[B46] Medeiros N. (2015). Global circumstances and recent trends in the Portuguese university publishing industry. *Anál. Soc*. 50, 582–603. doi: 10.31447/AS00032573.2015216.05

[B47] Méndez-Rátiva C. P. Córdoba-Restrepo J. F. Morales-Perdomo T. Torres-Torres I. Garzón-Forero D. A. (2025). Communication and scientific collaboration networks: a case study of the editorial universidad del rosario. Rev. Esp. Doc. Cient. 48:1600. doi: 10.3989/redc.2025.2.1600

[B48] Mihal I. (2021). University presses and the market. marketing trends at book fairs. Inf. Cult. Soc. 44, 117–142. doi: 10.34096/ics.i44.8215

[B49] Mihal I. (2024). Editorial translation around written culture: espacios para la lectura, a collection by fondo de cultura económica (1999-2003). mutatis mutandis. Rev. Latinoam. Trad. 17:2. doi: 10.17533/udea.mut.v17n2a08

[B50] Mihal I. Saferstein E. (2024). Books and mediatization on social networks: editorial communication strategies during the pandemic in Argentina. TSN Transatl. Stud. Netw. 16, 187–201. doi: 10.24310/tsn.16.2024.20208

[B51] Mihal I. Szpilbarg D. (2020). Prologue: university publishing and editorial policies as an object of analysis. *Cuad. Cent. Estud. Diseñ. Comun*. 85, 11–19. doi: 10.18682/cdc.vi85.3748

[B52] Olivares G. (2020). Presence of university presses in the calls for proposals of the Book Fund, Chile, 2013–2018. *Cuad. Cent. Estud. Diseñ. Comun*. 85, 95–106. doi: 10.18682/cdc.vi85.3754

[B53] Orensanz Escofet L. (2017). What kind of Spanish do we translate into in Mexico? The unity/diversity of the Spanish language according to a sample of Mexican translators. mutatis mutandis. Lat. Am. J. Transl. 10, 149–173. doi: 10.17533/udea.mut.v10n2a06

[B54] Restrepo-Arango C. (2023). Colombian university publishers. Rev. Ibero-Am. Cienc. Inf. 16, 402–423. doi: 10.26512/rici.v16.n2.2023.47330

[B55] Spinak E. (2018). Books' relevance in scholarly communication: the case of SciELO Books. SciELO Perspect. Available online at: https://blog.scielo.org/en/2018/08/07/books-relevance-in-scholarly-communication-the-case-of-scielo-books/ (Accessed May 12, 2026).

[B56] Szpilbarg D. (2020). Editorial policies and digitization: the case of EUDEBA and the digital reader “Boris”. *Cuad. Cent. Estud. Diseñ. Comun*. 85, 119–134. doi: 10.18682/cdc.vi85.3756

[B57] Tejada-Artigas C.-M. Giménez-Toledo E. Oliveira A. B. D. (2020). The prestige of the academic publishers with books in social sciences and humanities in Brazil. Transinform. 32:e190043. doi: 10.1590/2318-0889202032e190043

[B58] Torres-Salinas D. Robinson-García N. Cabezas-Clavijo Á. Jiménez-Contreras E. (2014). Analyzing the citation characteristics of books: edited books, book series and publisher types in the book citation index. Scientometrics 98, 2113–2127. doi: 10.1007/s11192-013-1168-4

[B59] Torres-Salinas D. Rodríguez-Sánchez R. Robinson-García N. Fdez-Valdivia J. García J. A. (2013). Mapping citation patterns of book chapters in the book citation index. J. Informetr. 7, 412–424. doi: 10.1016/j.joi.2013.01.004

[B60] UNESCO. (2021). UNESCO Recommendation on Open Science. Paris: United Nations Educational, Scientific and Cultural Organization. Available online at: https://unesdoc.unesco.org/ark:/48223/pf0000379949.locale=en (Accessed May 12, 2026).

[B61] Vázquez Álvarez I. (2021). The book and its industry, the state of play through analysis of its bibliographic production (1958-2021): perspectives and tools for mesoanalytical and mesoeconomic research. Gen. J. Inf. Doc. 31, 665–696. doi: 10.5209/rgid.79463

[B62] Vicente Romero C. H. (2021). Between springs and storms: the buenos aires university editorial in the violent beginning of the 1970s in Argentina. Rev. Eletrôn. ANPHLAC 21, 36–75. doi: 10.46752/anphlac.30.2021.3970

[B63] Zelarayán C. (2020). Crossroads of university publishing. *Cuad. Cent. Estud. Diseñ. Comun*. 85, 21–34. doi: 10.18682/cdc.vi85.3749

